# Atomic Model Structure of the NIST Monoclonal Antibody (NISTmAb) Reference Material

**DOI:** 10.6028/jres.126.012

**Published:** 2021-07-15

**Authors:** Christina Bergonzo, D. Travis Gallagher

**Affiliations:** 1 National Institute of Standards and Technology, Gaithersburg, MD 20899, USA

**Keywords:** intact antibody, molecular model, NISTmAb, RM8671

**Data DOI:** https://doi.org/10.18434/mds2-2396

## 1 Summary

As monoclonal antibodies have become a vital resource in medicine, knowledge of their complex molecular structures has increased in importance. Thousands of antibody components (Fab and Fc fragments) are described in the Protein Data Bank [1]. Whole antibodies have been imaged by electron microscopy methods [2, 3] and in a few cases, crystallized [4–6]. The central hinge lacks a unique stable conformation and its dynamic properties are important to antibody function. Monte Carlo and molecular dynamics simulations and small-angle scattering methods have been used to analyze the wide range of configurations that are accessible to antibodies in solution [7, 8]. In order to support the development of antibody-based medicines, the National Institute of Standards and Technology (NIST) has released an extensively characterized IgG1κ monoclonal antibody (mAb), called the NISTmAb Reference Material 8671^1^ [9–14]. To facilitate modeling of whole antibodies we now report the construction of an all-atom 3-D model of the NISTmAb.

^1^ Extensive information on NISTmAb is available at the websitenistmab.ibbr.umd.edu.

## 2 Data Specifications

**Table:** 

**NIST Operating Unit(s)**	MML, 645.03
**Format**	PDB Version 5.0 file format, FASTA sequence format
**Instrument**	N/A
**Spatial or Temporal Elements**	N/A
**Data Dictionary**	https://mmcif.wwpdb.org/dictionaries/mmcif_pdbx_v50.dic/Index/
**Accessibility**	All datasets submitted to *Journal of Research of NIST* are publicly available.
**License**	https://www.nist.gov/director/licensing

## 3 Methods

**Fig. 1:**
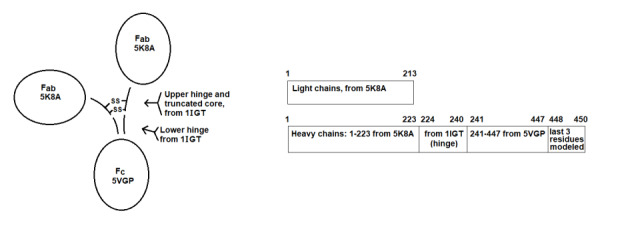
Origin of the NISTmAb model components by structure and by sequence. The diagram on the left shows how the model was assembled from 5 parts: two Fabs, one Fc, one Upper hinge with disulfide-containing core, and one Lower hinge. The coordinates were copied from three PDB crystal structures (5K8A[15], 5VGP[16], and 1IGT[4]) as indicated. Because the hinge is derived from crystal structure 1IGT, the conformation of the whole NISTmAb model inherits an asymmetric conformation similar to 1IGT. The schematic on the right gives the same information according to sequence. Full sequence information is available in a supplementary fasta-format sequence file.

**Fig. 2:**
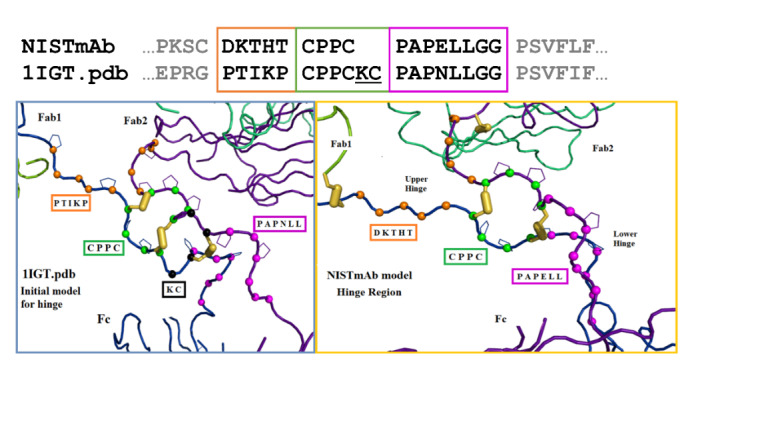
Top: Local alignment of hinge region for NISTmAb (residues 220 – 246) and 1IGT.pdb. Bottom, left: 1IGT.pdb hinge structure. Bottom, right: Final NISTmAb modeled hinge structure. Image made using PyMOL[17]

The NISTmAb antibody model was made from five parts as shown in Figure 1, using copies of its previously reported Fab (PDBID 5K8A [15]) and Fc (PDBID 5VGP [16]) fragment crystal structures. All parts of the hinge are derived from the whole-mAb crystal structure 1IGT [4], which uniquely includes the entire 4-Cys core, sequence CPPC, with both disulfides intact. The additional crosslink in 1IGT (its hinge has 3 disulfides) was removed to make NISTmAb, according to the hinge-region alignment in Figure 2 (top).

In order to obtain the correct upper and lower hinge lengths, the two underlined residues of 1IGT in Figure 2 (KC) were removed. The new gap between …CPPC and PAPN… was spliced together by moving the initial proline of PAPN… onto the location of the deleted lysine. Both heavy chains were spliced simultaneously. Then, appropriate mutations were made, using the rotamer library in PyMOL^2^ [17] to give the hinge model the correct NISTmAb sequence (FASTA format data file). Figure 2 shows the initial 1IGT hinge and the derived NISTmAb hinge.

^2^ Certain commercial equipment, instruments, or materials are identified in this paper to foster understanding. Such identification does not imply recommendation or endorsement by the National Institute of Standards and Technology, nor does it imply that the materials or equipment identified are necessarily the best available for the purpose.

**Fig. 3:**
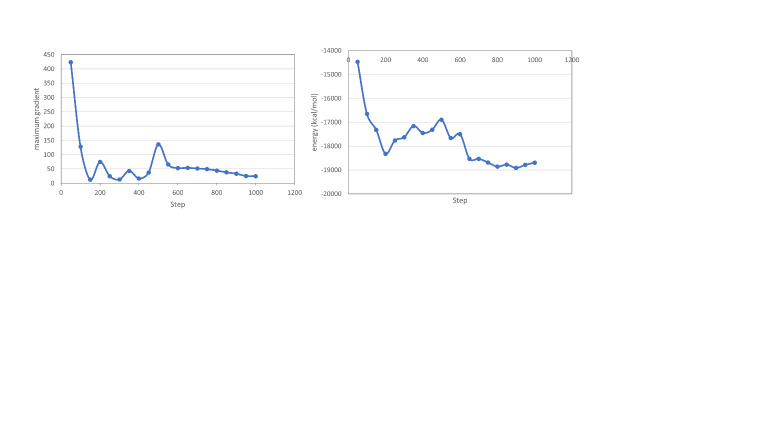
Left: Maximum energy gradient per step during minimization. Right: Energy per step during minimization.

The initial model was minimized to remove local strain introduced by splicing and to produce a refined, energy-minimized model with correct local geometry. The model was parameterized using the tLEaP program in Amber18 [18]. The ff14SB force field for proteins was used, and the glycan 06j-1 force field was used for glycans [19, 20]. Minimization was performed in vacuo for 1000 steps total, using the steepest descent algorithm for the first 500 steps before switching to the conjugate gradient algorithm for 500 steps. Plots of energy and maximum gradient vs. minimization step are shown in Figure 3.

The model includes all 1326 amino acids in four chains. The two light chains are designated as L and M. Both light chains have amino acids 1 to 213. The two heavy chains are designated as H and V chains and have amino acids 1 to 450. As in 5VGP, the G1F/G0F glycans, (i.e. the H-chain glycan has nine sugar groups including one fucose and one terminal galactose, while the V chain has only eight, lacking galactose) attach to Asn300. Thus, the H and V chains have identical amino acid sequence, but slightly different glycans. Each heavy chain has pyroglutamate (PCA) at the N-terminus, a commonly observed (at protein N-termini) conversion from the genetically encoded Glutamine, known to be present in the NISTmAb at near quantitative abundance [21]. In addition, while both chains in 5VGP lacked three C-terminal residues which were disordered in the Fc crystal structure, they are added to the model for the sake of completeness. The model includes all known atoms and has the composition given in Table 1.

**Table 1:** Summary of atoms comprising NISTmAb RM8671.

**Atom type**	**L/M chain**	**H/V chain**	**H+V glycans**	**Total**
C	1020	2218	118	6594
N	270	582	8	1712
O	330	674	83	2091
S	7	17	-	48
H	1574	3435	196	10214
**Total**	**3201**	**6926**	**405**	**20659**

**Fig. 4:**
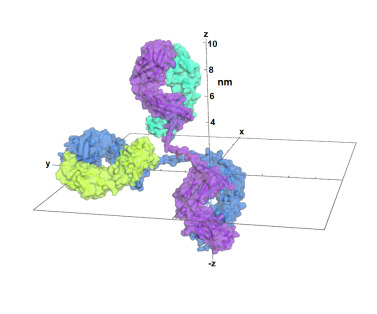

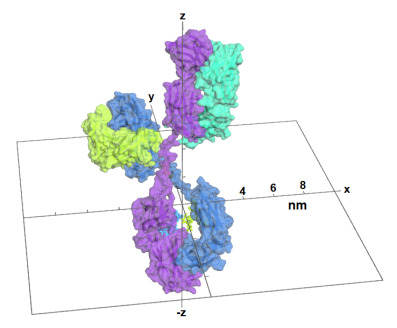
Two views of the NISTmAb model with cartesian reference frame. Heavy chains are colored blue and purple. Light chains and glycans are colored lime green and cyan. The protein is shown as a slightly transparent surface. The lowest part positioned below the xy plane (the Fc domain) has a molecular dyad that has been placed on the -z axis. Units along the axes show that each of the molecule’s 3 lobes extends about 10 nm from the origin. The view on the left shows the sharp bend in the paired heavy chains just above the origin. Image made using PyMOL [17]

To facilitate subsequent comparisons, the model has been oriented with the Fc dyad along the z-axis, placed with the xyz origin at the midpoint between the CA atoms of the two Proline 241 residues, at the top of the Fc. See Figure 4.

## 4 Impact

As the field of antibody therapeutics continues to grow, it is likely that structural and mechanistic models will serve increasingly to guide molecular design strategies. The reported model is intended to provide an all-atom structure that can be used as a reference for the interpretation of biophysical measurements and a starting point for further modeling and dynamics studies. Molecular dynamics simulation, together with continuing developments in metrology, are expected to lead to improved understanding of hinge properties, enabling better models of biological functions such as bivalent binding and signaling.

Hinge properties potentially involved in antibody function whose study may be facilitated by the present model include the sharp bend near the hinge C-terminus, at the GGP sequence 239-241. Crystal structures 1IGT [4], 1IGY [5], 1HZH [6] and 3AY4 [22] all have a similar bend, and in 3AY4 the bend appears necessary for Fc gamma receptor binding and thus effector signaling [22]. Additionally, the hinge core containing the paired CPPC motif forms a covalent ring of 28 atoms that includes two disulfides, the constrained nature of which could limit the conformational landscape of the Fab and Fc domains. Finally, the radius of gyration of this model, 5.6 nm, agrees closely with available solution state measured values for NISTmAb from scattering methods [7].
